# A hybrid parameter estimation algorithm for beta mixtures and applications to methylation state classification

**DOI:** 10.1186/s13015-017-0112-1

**Published:** 2017-08-18

**Authors:** Christopher Schröder, Sven Rahmann

**Affiliations:** Genome Informatics, Institute of Human Genetics, University of Duisburg-Essen, University Hospital Essen, Hufelandstr. 55, 45147 Essen, Germany

**Keywords:** Mixture model, Beta distribution, Maximum likelihood, Method of moments, EM algorithm, Differential methylation, Classification

## Abstract

**Background:**

Mixtures of beta distributions are a flexible tool for modeling data with values on the unit interval, such as methylation levels. However, maximum likelihood parameter estimation with beta distributions suffers from problems because of singularities in the log-likelihood function if some observations take the values 0 or 1.

**Methods:**

While ad-hoc corrections have been proposed to mitigate this problem, we propose a different approach to parameter estimation for beta mixtures where such problems do not arise in the first place. Our algorithm combines latent variables with the method of moments instead of maximum likelihood, which has computational advantages over the popular EM algorithm.

**Results:**

As an application, we demonstrate that methylation state classification is more accurate when using adaptive thresholds from beta mixtures than non-adaptive thresholds on observed methylation levels. We also demonstrate that we can accurately infer the number of mixture components.

**Conclusions:**

The hybrid algorithm between likelihood-based component un-mixing and moment-based parameter estimation is a robust and efficient method for beta mixture estimation. We provide an implementation of the method (“betamix”) as open source software under the MIT license.

## Background

The beta distribution is a continuous probability distribution that takes values in the unit interval [0, 1]. It has been used in several bioinformatics applications [1] to model data that naturally takes values between 0 and 1, such as relative frequencies, probabilities, absolute correlation coefficients, or DNA methylation levels of CpG dinucleotides or longer genomic regions. One of the most prominent applications is the estimation of false discovery rates (FDRs) from p-value distributions after multiple tests by fitting a beta-uniform mixture (BUM, [2]). By linear scaling, beta distributions can be used to model any quantity that takes values in a finite interval $[L,U]\subset \mathbb {R}$.

**Fig. 1:**
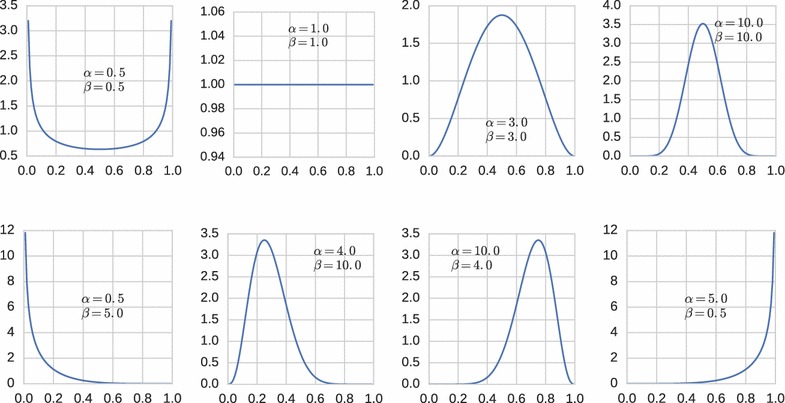
Different shapes of beta distributions depending on parameters $\alpha$ and $\beta$

The beta distribution has two parameters $\alpha >0$ and $\beta >0$ and can take a variety of shapes depending on whether $0<\alpha <1$ or $\alpha =1$ or $\alpha >1$ and $0<\beta <1$ or $\beta =1$ or $\beta >1$; see Fig.  1. The beta probability density on [0, 1] is

1 $$\begin{aligned} b_{\alpha ,\beta }(x) = \frac{1}{B(\alpha ,\beta )} \cdot x^{\alpha -1} \cdot (1-x)^{\beta -1} \,, \quad \text { where } B(\alpha ,\beta ) = \frac{\Gamma (\alpha )\Gamma (\beta )}{\Gamma (\alpha +\beta )} \,, \end{aligned}$$

and $\Gamma$ refers to the gamma function $\Gamma (z) = \int _0^\infty \, x^{z-1}\, \text {e}^{-x} \, \text {d}x$ with $\Gamma (n)=(n-1)!$ for positive integers *n*. It can be verified that $\int _0^1\, b_{\alpha ,\beta }(x) \,\text {d}x = 1$. For $\alpha =\beta =1$, we obtain the uniform distribution. Section “Preliminaries: Beta distributions” has more details.

While a single beta distribution can take a variety of shapes, *mixtures* of beta distributions are even more flexible. Such a mixture has the general form

2 $$\begin{aligned} f_\theta (x) = \sum _{j=1}^c\, \pi _j \cdot b_{\alpha _j,\beta _j}(x) \,, \end{aligned}$$

where *c* is the *number of components*, the $\pi _j$ are called *mixture coefficients* satisfying $\sum _j\, \pi _j=1$ and $\pi _j\ge 0$, and the $\alpha _j, \beta _j$ are called *component parameters*. Together, we refer to all of these as *model parameters* and abbreviate them as $\theta$. The number of components *c* is often assumed to be a given constant and not part of the parameters to be estimated.

The *parameter estimation problem* consists of estimating $\theta$ from *n* usually independent observed samples $(x_1,\dots ,x_n)$ such that the observations are well explained by the resulting distribution.

*Maximum likelihood* (ML) estimation (MLE) is a frequently used paradigm, consisting of the following optimization problem.

3 $$\begin{aligned} \text {Given } (x_1,\dots ,x_n),\; \text { maximize }&\mathcal {L}(\theta ) := \prod _{i=1}^n\, f_\theta (x_i), \nonumber \\ \text { or equivalently, }&L(\theta ) := \sum _{i=1}^n\, \ln f_\theta (x_i). \end{aligned}$$

As we show below in “Preliminaries: Maximum likelihood estimation for Beta distributions”, MLE has significant disadvantages for beta distributions. The main problem is that the likelihood function is not finite (for almost all parameter values) if any of the observed datapoints are $x_i=0$ or $x_i=1$.

For mixture distributions, MLE frequently results in a non-concave problem with many local maxima, and one uses heuristics that return a local optimum from given starting parameters. A popular and successful method for parameter optimization in mixtures is the expectation maximization (EM) algorithm [3] that iteratively solves an (easier) ML problem on each estimated component and then re-estimates which datapoints belong to which component. We review the basic EM algorithm below in the Section “Preliminaries: The EM algorithm for beta mixture distributions”.

Because already MLE for a single beta distribution is problematic, EM does not work for beta mixtures, unless ad-hoc corrections are made. We therefore propose a new algorithm for parameter estimation in beta mixtures that we call *iterated method of moments*. The method is presented in below in the Section “The iterated method of moments”.

Our main motivation for this work stems from the analysis of methylation level data in differentially methylated regions between individuals, not cell types or conditions; see Section “Application: classification of methylation states”. Our evaluation therefore focuses on the benefits of beta mixture modeling and parameter estimation using our algorithm for methylation state classification from simulated methylation level data.

## Preliminaries

### Beta distributions

The beta distribution with parameters $\alpha >0$ and $\beta >0$ is a continuous probability distribution on the unit interval [0, 1] whose density is given by Eq. (1).

If *X* is a random variable with a beta distribution, then its expected value $\mu$ and variance $\sigma ^2$ are

4 $$\begin{aligned} \mu := \mathbb {E}[X] = \frac{\alpha }{\alpha +\beta } \,, \quad \sigma ^2 := \text {Var}[X] = \frac{\mu (1-\mu )}{\alpha +\beta +1} = \frac{\mu (1-\mu )}{1+\phi } \,, \end{aligned}$$

where $\phi = \alpha +\beta$ is often called a *precision* parameter; large values indicate that the distribution is concentrated. Conversely, the parameters $\alpha$ and $\beta$ may be expressed in terms of $\mu$ and $\sigma ^2$: First, compute

5 $$\begin{aligned} \phi = \frac{\mu (1-\mu )}{\sigma ^2} - 1 \,; \quad \text {then}\quad \alpha = \mu \phi \,, \quad \beta = (1-\mu )\phi \,. \end{aligned}$$

The textbook by Karl Bury [4] has more details about moments and other properties of beta distributions and other distributions used in engineering.

### Maximum likelihood estimation for Beta distributions

The estimation of parameters in a parameterized distribution from *n* independent samples usually follows the maximum likelihood (ML) paradigm. If $\theta$ represents the parameters and $f_\theta (x)$ is the probability density of a single observation, the goal is to find $\theta ^*$ that maximizes $L(\theta )$ as defined in Eq. (3).

Writing $\gamma (y):= \ln \Gamma (y)$, the beta log-likelihood is

6 $$\begin{aligned} L(\alpha ,\beta ) = n (\gamma (\alpha +\beta ) - \gamma (\alpha ) - \gamma (\beta )) + (\alpha -1) \cdot \sum _i\, \ln x_i + (\beta -1) \cdot \sum _i \ln (1-x_i)\,. \end{aligned}$$

The optimality conditions $\text {d}L / \text {d}\alpha = 0$ and $\text {d}L / \text {d}\beta = 0$ must be solved numerically and iteratively because the parameters appear in the logarithm of the gamma function. In comparison to a mixture of Gaussians where analytical formulas exist for the ML estimators, this is inconvenient, but the main problem is a different one. The log-likelihood function is not well defined for $\alpha \ne 1$ if any of the observations are $x_i=0$, or for $\beta \ne 1$ if any $x_i=1$. Indeed, several implementations of ML estimators for beta distributions (e.g. the R package betareg, see below) throw errors then.

Note that, *in theory*, there is no problem, because $x\in \{0,1\}$ is an event of probability zero if the data are truly generated by a beta distribution. Real data, however, in particular observed methylation levels, may very well take these values. This article’s main motivation is the desire to work with observations of $x=0$ and $x=1$ in a principled way.

The above problem with MLE for beta distributions has been noted previously, but, to our knowledge, not explicitly attacked. We here discuss the work-arounds of which we are aware.

#### Reducing the interval

A typical ad-hoc solution is to linearly rescale the unit interval [0, 1] to a smaller sub-interval $[\varepsilon ,1-\varepsilon ]$ for some small $\varepsilon >0$ or to simply replace values $<\varepsilon$ by $\varepsilon$ and values $>1-\varepsilon$ by $1-\varepsilon$, such that, in both cases, the resulting adjusted observations are in $[\varepsilon ,1-\varepsilon ]$.

**Fig. 2:**
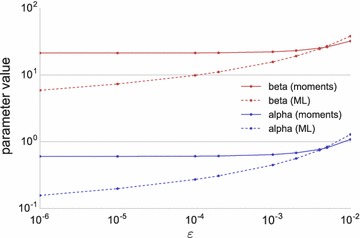
Estimated parameter values $\alpha$ (*blue*) and $\beta$ (*red*) from a dataset consisting of the ten observations $0.01, \dots , 0.10$ and 10 observations of $\varepsilon$ for varying values of $\varepsilon$. Estimation was done using MLE (*dotted lines*) as implemented in the R package betareg and by our (moment-based) method (*solid lines*).

A simple example, which has to our knowledge not been presented before, will show that the resulting parameter estimates depend strongly on the choice of $\varepsilon$ in the ML paradigm. Consider 20 observations, 10 of them at $x=0$, the remaining ten at $x=0.01, \dots , 0.10$. For different values of $0<\varepsilon <0.01$, replace the ten zeros by $\varepsilon$ and compute the ML estimates of $\alpha$ and $\beta$. We used the R package betareg 1 [5], which performs numerical ML estimation of $\text {logit}(\mu )$ and $\ln (\phi )$, where $\text {logit}(\mu ) = \ln (\mu /(1-\mu ))$. We then used Eq. (5) to compute ML estimates of $\alpha$ and $\beta$. We additionally used our iterated method of moments approach (presented in the remainder of this article) with the same varying $\varepsilon$. In contrast to MLE, our approach also works with $\varepsilon =0$. The resulting estimates for $\alpha$ and $\beta$ are shown in Fig. 2: not only is our approach able to directly use $\varepsilon =0$; it is also insensitive to the choice of $\varepsilon$ for small $\varepsilon >0$.

#### Using a different objective function

MLE is not the only way to parameter estimation. A more robust way for beta distributions may be to consider the cumulative distribution function (cdf) $F_\theta (x) := \int _0^x\, f_\theta (y)\, \text {d}y$ and compare it with the empirical distribution function $\hat{F}(x)$, the fraction of observations $\le x$. One can then choose the parameters $\theta$ such that a given distance measure between these functions, such as the Kolmogorov–Smirnov distance

7 $$\begin{aligned} d_\text {KS}(F_\theta ,\hat{F}) := \max _x\, |F_\theta (x) - \hat{F}(x)| \end{aligned}$$

is minimized. This optimization has to be performed numerically. We are not aware of specific implementations of this method for beta distributions or beta mixtures. In this work, we opted for a more direct approach based on the density function.

#### Using explicit finite-sample models

As we stated above, in theory, observations of $X=0$ or $X=1$ happen with probability zero if *X* has a continuous beta distribution. These observations do happen in reality because either the beta assumption is wrong, or we neglected the fact that the observation comes from a finite-precision observation. For methylation level data, the following model may be a more accurate representation of the data: To obtain a given datapoint $x_i$, first choose the true methylation level $p_i$ from the beta distribution with parameters $\alpha , \beta$. Then choose the observation $x_i$ from the binomial distribution with success probability $p_i$ and sample size $n_i$. The parameter $n_i$ controls the granularity of the observation, and it may be different for each *i*. In our application setting, $p_i$ would be the true methylation level of a specific CpG dinucleotide in individual *i*, and $x_i$ would be the observed methylation level with sequencing coverage $n_i$. This richer model captures the relationships between parameters and observations much better, but the estimation process also becomes more complex, especially if the $n_i$ are not available.

#### Summary

While MLE is known to be statistically efficient for correct data, its results may be sensitive to perturbations of the data. For modeling with beta distributions in particular, the problems of MLE are severe: The likelihood function is not well defined for reasonable datasets that occur in practice, and the solution depends strongly on ad-hoc parameters introduced to rectify the first problem. Alternative models turn out to be computationally more expensive. Before we can introduce our solution to these problems, we first discuss parameter estimation in mixture models.

### The EM algorithm for beta mixture distributions

For parameters $\theta$ of mixture models, including each component’s parameters and the mixture coefficients, the log-likelihood function $L(\theta ) = \sum _{i=1}^n\, \ln f_\theta (x_i)$, with $f_\theta (x_i)$ as in Eq. (2), frequently has many local maxima; and a globally optimal solution is difficult to compute.

The EM algorithm [3] is a general iterative method for ML parameter estimation with incomplete data. In mixture models, the “missing” data is the information which sample belongs to which component. However, this information can be estimated (given initial parameter estimates) in the E-step (expectation step) and then used to derive better parameter estimates by ML for each component separately in the M-step (maximization step). Generally, EM converges to a local optimum of the log-likelihood function [6].

#### E-step

To estimate the expected responsibility $W_{i,j}$ of each component *j* for each data point $x_i$, the component’s relative probability at that data point is computed, such that $\sum _j\, W_{i,j} = 1$ for all *i*. Averaged responsibility weights yield new mixture coefficients $\pi ^+_j$.

8 $$\begin{aligned} W_{i,j} = \frac{ \pi _j\, b_{\alpha _j,\beta _j}(x_i)}{ \sum _k\, \pi _k\, b_{\alpha _k,\beta _k}(x_i)} \, \quad \text {and}\quad \pi ^+_j = \frac{1}{n} \sum _{i=1}^n\, W_{i,j} \,. \end{aligned}$$

#### M-step

Using the responsibility weights $W_{i,j}$, the components are unmixed, and a separate (weighted) sample is obtained for each component, so their parameters can be estimated independently by MLE. The new mixture coefficients’ ML estimates $\pi ^+_j$ in Eq. (8) are indeed the averages of the responsibility weights over all samples.

#### Initialization and termination

EM requires initial parameters before starting with an E-step. The resulting local optimum depends on these initial parameters. It is therefore common to choose the initial parameters either based on additional information (e.g., one component with small values, one with large values), or to re-start EM with different random initializations. Convergence is detected by monitoring relative changes among the log-likelihood or among parameters between iterations and stopping when these changes are below a given tolerance.

#### Properties and problems with beta mixtures

One of the main reasons why the EM algorithm is predominantly used in practice for mixture estimation is the availability of an objective function (the log-likelihood). By Jensen’s inequality, it increases in each EM iteration, and when it stops increasing, a stationary point has been reached [6]. Locally optimal solutions obtained by two runs with different initializations can be objectively and globally compared by comparing their log-likelihood values.

In beta mixtures, there are several problems with the EM algorithm. First, the responsibility weights $W_{i,j}$ are not well defined for $x_i=0$ or $x_i=1$ because of the singularities in the likelihood function, as described above. Second, the M-step cannot be carried out if the data contains any such point for the same reason. Third, even if all $x_i\in \, ]0,1[$, the resulting mixtures are sensitive to perturbations of the data. Fourth, because each M-step already involves a numerical iterative maximization, the computational burden over several EM iterations is significant. We now propose a computationally lightweight algorithm for parameter estimation in beta mixtures that does not suffer from these drawbacks.

## The iterated method of moments

With the necessary preliminaries in place, the main idea behind our algorithm can be stated briefly before we discuss the details.

From initial parameters, we proceed iteratively as in the EM framework and alternate between an E-step, which is a small modification of EM’s E-step, and a parameter estimation step, which is not based on the ML paradigm but on Pearson’s method of moments until a stationary point is reached [7].

To estimate *Q* free parameters, the method of moments’ approach is to choose *Q* moments of the distribution, express them through the parameters and equate them to the corresponding *Q* sample moments. This usually amounts to solving a system of *Q* non-linear equations. In simple cases, e.g., for expectation and variance of a single Gaussian distribution, the resulting estimates agree with the ML estimates. Generally, this need not be the case.

The method of moments has been applied directly to mixture distributions. For example, a mixture of two one-dimensional Gaussians has $Q=5$ parameters: two means $\mu _1, \mu _2$, two variances $\sigma _1^2, \sigma _2^2$ and the weight $\pi _1$ of the first component. Thus one needs to choose five moments, say $m_k := \mathbb {E}[X^k]$ for $k=1,\dots ,5$ and solve the corresponding relationships. Solving these equations for many components (or in high dimensions) seems daunting, even numerically. Also it is not clear whether there is always a unique solution.

For a single beta distribution, however, $\alpha$ and $\beta$ are easily estimated from sample mean and variance by Eq. (5), using sample moments instead of true values. Thus, to avoid the problems of MLE in beta distributions, we replace the likelihood maximization step (M-step) in EM by a method of moments estimation step (MM-step) using expectation and variance.

We thus combine the idea of using latent responsibility weights from EM with moment-based estimation, but avoid the problems of pure moment-based estimation (large non-linear equation systems). It may seem surprising that nobody appears to have done this before, but one reason may be the lack of an objective function, as we discuss further below.

### Initialization

A general reasonable strategy for beta mixtures is to let each component focus on a certain sub-interval of the unit interval. With *c* components, we start with one component responsible for values around $k/(c-1)$ for each $k=0,\dots ,c-1$. The expectation and variance of the component near $k/(c-1)$ is initially estimated from the corresponding sample moments of all data points in the interval $[(k-1)/(c-1), (k+1)/(c-1)] \cap [0,1]$. (If an interval contains no data, the component is removed from the model.) Initial mixture coefficients are estimated proportionally to the number of data points in that interval.

A second common strategy are randomized start parameters. Instead of using purely uniform random choices, more advanced methods are available, e.g. the $D^2$-weighted initialization used by k-means++ [8]. We here adapted this idea. Let $X \subset [0,1]$ be the set of different data values. Let $Y \subset X$ be the set of chosen component centers, initially $Y=\{\}$. Let $D_Y(x) := \min _{y\in Y}\, |x-y|$ be the shortest distance of *x* to any already chosen data point. The initialization then consists of the following steps.

1. Choose the first point *y* uniformly at random from *X*; set $Y:=\{y\}$.
2. Repeat until $|Y|=c$: Choose $y\in X\setminus Y$ with probability proportional to $D_Y(y)^2$; then set $Y := Y \cup \{y\}$.
3. Sort *Y* such that $y_1< \dots < y_c$.
4. Expectation and variance of component $j=1,\dots , c$ are initially estimated from the corresponding sample moments of all data points in the interval $[y_j-0.5,\, y_j+0.5]$.

EM-like algorithms are usually repeatedly executed with different random initializations, and the parameters with the best locally optimal log-likelihood are finally returned as the result.

### E-step

The E-step is essentially the same as for EM, except that we assign weights explicitly to data points $x_i=0$ and $x_i=1$.

Let $j_0$ be the component index *j* with the smallest $\alpha _j$. If there is more than one, choose the one with the largest $\beta _j$. The $j_0$ component takes full responsibility for all *i* with $x_i=0$, i.e., $W_{i,j_0}=1$ and $W_{i,j}=0$ for $j\ne j_0$. Similarly, let $j_1$ be the component index *j* with the smallest $\beta _j$ (among several ones, the one with the largest $\alpha _j$). For all *i* with $x_i=1$, set $W_{i,j_1}=1$ and $W_{i,j}=0$ for $j\ne j_1$.

### MM-step

The MM-step estimates mean and variance of each component *j* by responsibility-weighted sample moments,

9 $$\begin{aligned} \mu _j = \frac{ \sum _{i=1}^n\, W_{ij} \cdot x_i}{ \sum _{i=1}^n\, W_{ij}} = \frac{ \sum _{i=1}^n\, W_{ij} \cdot x_i}{ n\cdot \pi _j}, \qquad \sigma ^2_j = \frac{ \sum _{i=1}^n\, W_{ij} \cdot (x_i - \mu _j)^2}{ n\cdot \pi _j} \,. \end{aligned}$$

Then $\alpha _j$ and $\beta _j$ are computed according to Eq. (5) and new mixture coefficients according to Eq. (8).

### Termination

Let $\theta _q$ be any real-valued parameter to be estimated and $T_q$ a given threshold for $\theta _q$. After each MM-step, we compare $\theta _q$ (old value) and $\theta ^+_q$ (updated value) by the relative change $\kappa _{q} := |\theta _q^+ - \theta _{q}|/{\max}\left( |\theta _{q}^+|,|\theta _{q}| \right)$. (If $\theta _{q}^+ = \theta _{q} = 0$, we set $\kappa _{q} := 0$.) We say that $\theta _q$ is stationary if $\kappa _q < T_q$. The algorithm terminates when all parameters are stationary.

### Properties

The proposed hybrid method does not have a natural objective function that can be maximized. Therefore we cannot make statements about improvement of such a function, nor can we directly compare two solutions from different initializations by objective function values. It also makes no sense to talk about “local optima”, but, similar to the EM algorithm, there may be several stationary points. We have not yet established whether the method always converges. On the other hand, we have the following desirable property.

#### **Lemma 1**

*In each MM-step, before updating the component weights, the expectation of the estimated density equals the sample mean. In particular, this is true at a stationary point.*

#### Proof

For a density *f* we write $\mathbb {E}[f]$ for its expectation $\int x \cdot f(x) \, \text {d}x$. For the mixture density (2), we have by linearity of expectation that $\mathbb {E}[f_\theta ] = \sum _j\, \pi _j \, \mathbb {E}[b_{\alpha _j,\beta _j}] = \sum _j\, \pi _j\, \mu _j$. Using (9) for $\mu _j$, this is equal to $\frac{1}{n} \sum _j\, \sum _i\, W_{ij}\, x_i = \frac{1}{n} \sum _i\, x_i$, because $\sum _j\, W_{ij}=1$ for each *j*. Thus $\mathbb {E}[f_\theta ]$ equals the sample mean. $\square$

Different objective functions may be substituted for the log-likelihood to compare different stationary points, such as the previously mentioned Kolmogorov–Smirnov distance $d_\text {KS}$ from Eq. (7). While we do not use it for optimization directly (our approach is more lightweight), we can use it to evaluate different stationary points and to estimate the number of necesssary components to represent the data.

### Estimating the number of components

The method described so far works for a given and fixed number of components, similarly to the EM algorithm. When the true number of components is unknown, the algorithm has to estimate this number by comparing goodness of fit between the estimated beta mixture and the given data, taking into account the model complexity (number of parameters). Usually the Akaike information criterion (AIC) [9] or Bayesian information criterion (BIC) [10] are minimized for this purpose,

10 $$\begin{aligned} AIC = 2k - 2 L^*, \quad BIC = k \ln (n) -2 L^* \end{aligned}$$

where $L^*$ is the maximized log-likelihood value, *k* is the number of free model parameters and *n* is the sample size. Both criteria favor a good fit but penalize many parameters (complex models with many components). Since our approach is not based on likelihoods, we cannot apply these criteria.

Instead, we use the Kolmogorov–Smirnov distance $d_\text {KS}$ from Eq. (7) to measure the fit between the estimated mixture cumulative distribution function (cdf), evaluated numerically at each data point, and the empirical cumulative distribution function from the data. Naturally, $d_\text {KS}$ is a decreasing function of the number of components. We fit models with an increasing number of components and stop once $d_\text {KS}$ drops below a given threshold. Note that for fixed sample size *n*, the distance $d_\text {KS}$ can be converted into a p-value of the Kolmogorov–Smirnov test and vice versa [11].

## Application: classification of methylation states

### Motivation

We are interested in explaining differences in methylation levels of genomic regions between individuals by genetic variation and would like to find single nucleotide variants (SNVs) whose state correlates well with methylation state. In a diploid genome, we expect the methylation level of a homogeneously methylated region in a homogeneous collection of cells to be (close to) 0, 0.5 or 1, and the state of the corresponding region may be called unmethylated, semi-methylated or fully methylated, respectively.

When we measure the methylation level of each CpG dinucleotide in the genome, for example by whole genome bisulfite sequencing (WGBS) [12], we observe fractions $M/(M+U)$ from numbers *M* and *U* of reads that indicate methylated and unmethylated cytosines, respectively, at each CpG dinucleotide. These observed fractions differ from the true methylation levels for several reasons: incomplete bisulfite conversion, sequencing errors, read mapping errors, sampling variance due to a finite number of reads, an inhomogeneous collection of cells being sequenced, the region being heterogeneously methylated, and others.

Therefore we model the observed methylation level by a probability distribution depending on the methylation state. The overall distribution of the observations is captured by a three-component beta mixture model with one component representing values close to zero (unmethylated), one component close to 1/2 (semi-methylated), and one component close to 1 (fully methylated).

Thus the problem is as follows. After seeing *n* observed methylation levels $(x_1,\dots ,x_n)$, find the originating methylation state for each $x_i$. This is frequently done using reasonable fixed cut-off values (that do not depend on the data), e.g. calling values below 0.25 unmethylated, values between 0.25 and 0.75 semi-methylated and values above 0.75 fully methylated [13]. One may leave $x_i$ unassigned if the value is too close to one of the cut-off values.

An interesting question is whether choosing thresholds adaptively based on the observed sample is advantageous in some sense. Depending on the components’ parameters, the value range of the components may overlap, and perfect separation may not be possible based on the value of $x_i$. Good strategies should be based on the component weights $W_{ij}$, assigning component $j^*(i) :=$ $argmax_j$  $W_{ij}$ to $x_i$. We may refuse to make an assignment if there is no clearly dominating component, e.g., if $W^*_i := \max _j\, W_{ij} < T$, or if $W^*_i - W^{(2)}_i < T$ for a given threshold *T*, where $W^{(2)}_i$ is the second largest weight among the $W_{ij}$.

### Simulation and fitting for class assignment

We investigate the advantages of beta mixture modeling by simulation. In the following, let *U* be a uniform random number from [0, 1].

We generate two datasets, each consisting of 1000 three-component mixtures. In the first (second) dataset, we generate 200 (1000) samples per mixture.

To generate a mixture model, we first pick mixture coefficients $\pi =(\pi _1,\pi _2,\pi _3)$ by drawing $U_1, U_2, U_3$, computing $s:=\sum _j\, U_j$ and setting $\pi _j := U_j/s$. This does not generate a uniform element of the probability simplex, but induces a bias towards distributions where all components have similar coefficients, which is reasonable for the intended application. The first component represents the unmethylated state; therefore we choose an $\alpha \le 1$ and a $\beta >1$ by drawing $U_1, U_2$ and setting $\alpha := U_1$ and $\beta := 1/U_2$. The third component represents the fully methylated state and is generated symmetrically to the first one. The second component represents the semi-methylated state (0.5) and should have large enough approximately equal $\alpha$ and $\beta$. We draw $U_1, U_2$ and define $\gamma := 5/{\min}\{U_1,U_2\}$. We draw *V* uniformly between 0.9 and 1.1 and set $\alpha := \gamma V$ and $\beta := \gamma /V$.

To draw a single random sample *x* from a mixture distribution, we first draw the component *j* according to $\pi$ and then value *x* from the beta distribution with parameters $\alpha _j, \beta _j$. After drawing $n=200$ (dataset 1) or $n=1000$ (dataset 2) samples, we modify the result as follows. For each mixture sample from dataset 1, we set the three smallest values to 0.0 and the three largest values to 1.0. In dataset 2, we proceed similarly with the 10 smallest and largest values.

We use the algorithm as described above to fit a three component mixture model, with a slightly different initialization. The first component is estimated from the samples in [0, 0.25], the second one from the samples in [0.25, 0.75] and the third one from the samples in [0.75, 1]. The first (last) component is enforced to be falling (rising) by setting $\alpha _1=0.8$ ($\beta _3=0.8$) if it is initially estimated larger.

**Fig. 3:**
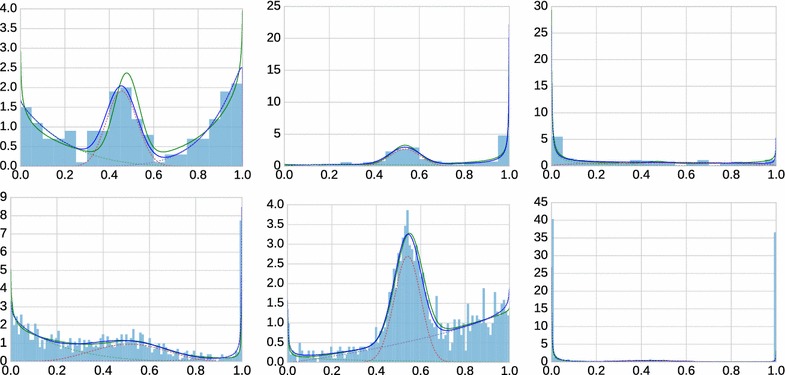
Examples of generated three-component beta mixtures (*green solid lines*), data samples (*blue histograms*) and fitted mixture models (*blue solid lines*). *Dashed lines* show estimated weighted component densities (*green*: unmethylated; *red*: semi-methylated; *magenta*: fully methylated). *Top row*: examples with $n=200$ samples; *bottom row*: $n=1000$

Figure 3 shows examples of generated mixture models, sampled data and fitted models. The examples have been chosen to convey a representative impression of the variety of generated models, from well separated components to close-to-uniform distributions in which the components are difficult to separate. Overall, fitting works well (better for $n=1000$ than for $n=200$), but our formal evaluation concerns whether we can infer the methylation state.

### Evaluation of class assignment rules

Given the samples $(x_1,\dots ,x_n)$ and the information which component $J_i$ generated which observation $x_i$, we evaluate different procedures:

1. Fixed intervals with a slack parameter $0\le s\le 0.25$: point *x* is assigned to the leftmost component if $x\in [0, 0.25-s]$, to the middle component if $x\in ]0.25+s, 0.75-s]$ and to the right component if $x\in ]0.75+s,1]$. The remaining points are left unassigned. For each value of *s*, we obtain the number of assigned points *N*(*s*) and the number of correctly assigned points $C(s)\le N(s)$. We plot the fraction of correct points *C*(*s*)/*n* and the precision *C*(*s*)/*N*(*s*) against the fraction of assigned points *N*(*s*)/*n* for different $s\ge 0$.
2. Choosing the component with the largest responsibility weight, ignoring points when the weight is low: point $x_i$ is assigned to component $j^*$ with maximal responsibility $W^*_i = W_{ij^*}$, unless $W_{ij^*}<t$ for a given threshold $0\le t\le 1$, in which case it is left unassigned. We examine the resulting numbers *C*(*t*) and *N*(*t*) as for the previous procedure.
3. Choosing the component with the largest responsibility weight, ignoring points when the distance to the second largest weight is low: as before, but we leave points $x_i$ unassigned if they satisfy $W_i^* - W^{(2)}_i < t$.
4. Repeating 2. and 3. with the EM algorithm instead of our algorithm would be interesting, but for all reasonable choices of $\varepsilon$ (recall that we have to replace $x_i=0$ by $\varepsilon$ and $x_i=1$ by $1-\varepsilon$ for EM to have a well-defined log-likelihood function), we could not get the implementation in betareg to converge; it exited with the message “no convergence to a suitable mixture”.

**Fig. 4:**
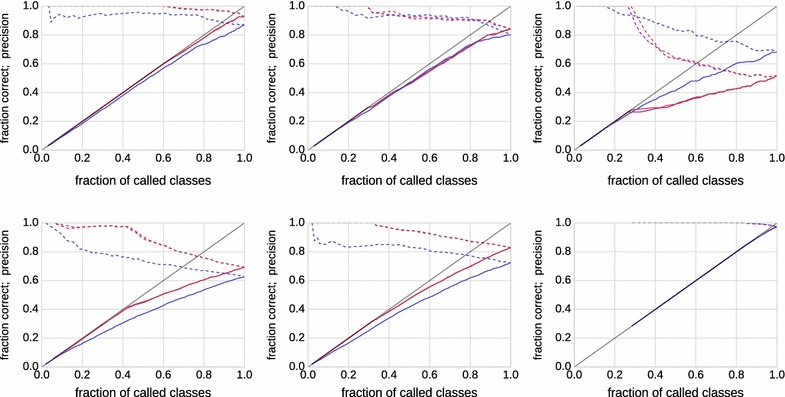
Performance of several classification rules. Shown is the fraction of called classes *N*/*n* (i.e., data points for which a decision was made) on the x-axis against the fraction of correct classes *C*/*n* (*solid lines*) and against the precision *C*/*N* (*dashed lines*) on the y-axis for three decision rules (*blue*: fixed intervals; *red*: highest weight with weight threshold; *magenta*: highest weight with gap threshold). The datasets are in the same layout as in Fig. 3

Figure 4 shows examples (the same as in Fig. 3) of the performance of each rule (rule 1: blue; rule 2: red; rule 3: magenta) in terms of *N*/*n* against *C*/*n* (fraction correct: solid) and *C*/*N* (precision: dashed). If a red or magenta curve is predominantly above the corresponding blue curve, using beta mixture modeling is advantageous for this dataset. Mixture modeling *fails* in particular for the example in the upper right panel. Considering the corresponding data in Fig.  3, the distribution is close to uniform except at the extremes, and indeed this is the prototypical case where beta mixtures do more harm than they help.

**Fig. 5:**
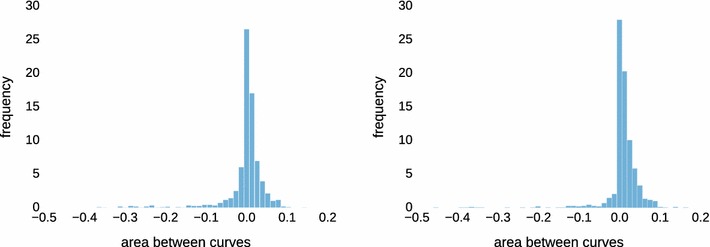
Signed areas between the *red curve* and the *blue curve* in Fig. 4 for all 1000 simulated mixtures in dataset 1 (*left*; 200 samples each) and in dataset 2 (*right*; 1000 samples each)

We are interested in the average performance over the simulated 1000 mixtures in dataset 1 ($n=200$) and dataset 2 ($n=1000$). As the magenta and red curve never differed by much, we computed the (signed) area between the solid red and blue curve in Fig. 4 for each of the 1000 mixtures. Positive values indicate that the red curve (classification by mixture modeling) is better. For dataset 1, we obtain a positive sign in 654/1000 cases (+), a negative sign in 337/1000 cases (−) and absolute differences of at most $10^{-6}$ in 9/1000 cases (0). For dataset 2, the numbers are 810/1000 (+), 186/1000 (−) and 4/1000 (0). Figure 5 shows histograms of the magnitudes of the area between curves. While there are more instances with benefits for mixture modeling, the averages ($-0.0046$ for dataset 1; $+0.0073$ for dataset 2) do not reflect this because of a small number of strong outliers on the negative side. Without analyzing each instance separately here, we identified the main cause for this behavior as close-to-uniformly distributed data, similar to the example in the upper right panel in Figs. 3 and 4, for which appropriate (but incorrect) parameters are found. In fact, a single beta distribution with $\alpha <0$ and $\beta <0$ would fit that data reasonably well, and the three-component model is not well identifiable. Of course, such a situation can be diagnosed by computing the distance between the sample and uniform distribution, and one can fall back to fixed thresholds.

### Simulation and fitting for estimating the number of components

To evaluate the component estimation algorithm, we simulate datasets with one to five components with $n=1000$ samples. We simulate two different kinds of datasets, both using the method of picking the mixture coefficients $\pi$ as described before.

#### Independent simulation

For the dirst kind of data, we choose components independently from each other. This frequently leads to datasets that can be effectively described by fewer components than the number used to generate the dataset. Let *E* be a standard exponentially distributed random variable with density function $f(x) = e^{-x}$. The parameters are chosen for each component *j* independently by choosing $\alpha = E_{j,1}$ and $\beta = 1-E_{j,2}$ from independent exponentials. (If $\beta <0$, we re-draw.)

#### Realistic simulation

We simulate more realistic and separable data by a second approach. The intention is to generate mixtures whose components are approximately equally distributed on the unit interval, such that each component slightly overlaps with its neighbors.

To generate a set of data points we pick an interval $I = [E_1, 1 - E_2]$ with exponentially distributed borders. (If $1-E_2 < E_1$, or if the interval is too small to admit *c* components with sufficient distance from each other, we re-draw.) For each component *j* we uniformly choose a point $\mu _j \in I$. We repeat this step if the distance between any two $\mu$ values is smaller than 0.2. Sort the values such that $E_1< \mu _1< \dots< \mu _c < 1-E_2$. Let $d_j := \min [\{|\mu _i-\mu _j| : i\ne j\}\ \cup \{E_1, 1-E_2\}]$. Then we set $\sigma _j = 1/4 d_j$. Now $\mu$ and $\sigma$ serve as mean and standard deviation for each component to generate its parameters $\alpha _j$ and $\beta _j$ by Eq. (5).

### Evaluation of component estimation

**Fig. 6:**
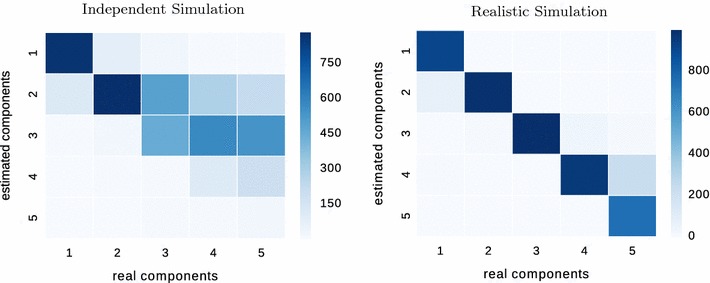
Comparison of the real number of components (*x*-axis) and the estimated number of components (*y*-axis) by our algorithm. Simulations consisted of 1000 datasets with 1000 data points each.* Each column* of each matrix sums to 1000; row sums are variable

We estimate the number of components as described above with a $d_\text {KS}$ threshold corresponding to a p-value of $\ge 0.5$ of the corresponding Kolmogorov–Smirnov test (as the fit becomes better with more components, the p-value is increasing). (The choice of 0.5 as a p-value threshold is somewhat arbitrary; it was chosen because it shows that there is *clearly no* significant deviation between the fitted mixture and the empirical cdf from the data; see below for the influence of this choice.) We compare the true simulated number of components to the estimated number for 1000 datasets of 1000 points each, generated by (a) independent simulation and (b) realistic simulation. Figure 6 shows the resulting confusion matrix. Near-perfect estimation would show as a strong diagonal. We see that we under-estimate the number of components on the independently generated data, especially for higher numbers of components. This is expected since the components of the independent simulation often overlap and result in relatively flat mixture densities that cannot be well separated. For the data from the realistic stimualtions, we can see a strong diagonal: Our algorithm rarely over- or underestimates the number of components if the components are separable. For both kinds of datasets, our method rarely overestimates the number of components.

**Fig. 7:**
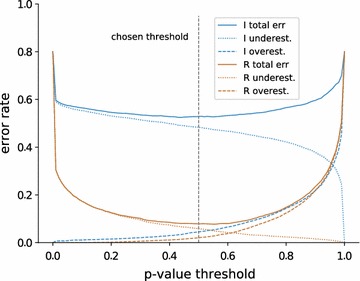
Fraction of under- and overestimations and total error rate (their sum) for datasets “independent” (I; *blue*) and “realistic” (R; *brown*) for varying p-value threshold of the Kolmogorov–Smirnov stopping criterion when choosing the number of mixture components

#### Choice of p-value threshold

In principle, we can argue for any “non-significant” p-value threshold. Choosing a low threshold would yield mixtures with fewer components, hence increase underestimations but reduce overestimations. Choosing a high threshold would do the opposite. By systematically varying the threshold we can examine whether there is an optimal threshold, maximizing the number of correct component estimations. Figure 7 shows the fraction of both under- and overestimations for both datasets (I: independent, blue; R: realistic, brown), as well as the total error rate (sum of under- and overestimation rates) for varying p-value threshold. We see that the error rate is generally higher in the independent model (I) because we systematically underestimate the true number of components (see above); this is true for any reasonable threshold $\le$ 0.9. We also see that both total error curves have a flat valley between 0.4 and 0.6 (or even 0.2 and 0.8), so choosing any threshold in this range is close to optimal; we chose 0.5 because it is “least complex” in the sense of Occam’s Razor.

## Discussion and conclusion

Maximum likelihood estimation in beta mixture models suffers from two drawbacks: the inability to directly use 0/1 observations, and the sensitivity of estimates to ad-hoc parameters introduced to mitigate the first problem. We presented an alternative parameter estimation algorithm for mixture models. The algorithm is based on a hybrid approach between maximum likelihood (for computing responsibility weights) and the method of moments; it follows the iterative framework of the EM algorithm. For mixtures of beta distributions, it does not suffer from the problems introduced by ML-only methods. Our approach is computationally simpler and faster than numerical ML estimation in beta distributions. Although we established a desirable invariant of the stationary points, other theoretical properties of the algorithm remain to be investigated. In particular, how can stationary points be characterized?

With a simulation study based on realistic parameter settings, we showed that beta mixture modeling is often beneficial when attempting to infer an underlying single nucleotide variant state from observed methylation levels, in comparison to the standard non-adaptive threshold approach. Mixture modeling failed when the samples were close to a uniform distribution without clearly separated components. In practice, we can detect such cases before applying mixture models and fall back to simple thresholding.

We also showed that for reasonably separated components, our method often infers the correct number of components. As the log-likelihood is not available for comparing different parameter sets (the value would be $\pm \infty$), we used the surrogate Kolmogorov–Smirnov (KS) distance between the estimated cumulative distribution function (cdf) and the empirical cdf. We showed that using any p-value threshold close to 0.5 for the corresponding KS test yields both good and robust results. Under-estimation is common if the data has low complexity (flat histograms) and can be effectively described with fewer components.

A comparison of our algorithm with the EM algorithm (from the betareg package) failed because the EM algorithm did not converge and exited with errors (however, we did not attempt to provide our own implementation). We hope that our method will be widely adopted in the future for other problems involving beta mixtures because of its computational advantages, and we intend to further characterize its properties.

## Footnotes

1
https://cran.r-project.org/web/packages/betareg/betareg.pdf.
